# Protective Effects of Zerumbone on Colonic Tumorigenesis in Enterotoxigenic *Bacteroides fragilis* (ETBF)-Colonized AOM/DSS BALB/c Mice

**DOI:** 10.3390/ijms21030857

**Published:** 2020-01-29

**Authors:** Soonjae Hwang, Minjeong Jo, Ju Eun Hong, Chan Oh Park, Chang Gun Lee, Ki-Jong Rhee

**Affiliations:** 1 Department of Biomedical Laboratory Science, College of Health Sciences, MIRAE Campus, Yonsei University, Wonju, Gangwon-do 26493, Korea; yoyosong1@naver.com (S.H.); minjeongjo12@gmail.com (M.J.); raperm87@gmail.com (J.E.H.); cksdh9453@gmail.com (C.O.P.); dangsunsang@naver.com (C.G.L.); 2 Cell Therapy and Tissue Engineering Center, Wonju College of Medicine, Yonsei University, Wonju, Gangwon-do 26426, Korea

**Keywords:** zerumbone, enterotoxigenic *Bacteroides fragilis*, colorectal cancer, colitis, polyps

## Abstract

Chronic inflammation has been linked to colitis-associated colorectal cancer in humans. The human symbiont enterotoxigenic *Bacteroides fragilis* (ETBF), a pro-carcinogenic bacterium, has the potential to initiate and/or promote colorectal cancer. Antibiotic treatment of ETBF has shown promise in decreasing colonic polyp formation in murine models of colon cancer. However, there are no reported natural products that have shown efficacy in decreasing polyp burden. In this study, we investigated the chemopreventive effects of oral administration of zerumbone in ETBF-colonized mice with azoxymethane (AOM)/dextran sulfate sodium (DSS)-induced tumorigenesis. Zerumbone significantly reduced the severity of disease activity index (DAI) scores as well as several parameters of colonic inflammation (i.e., colon weight, colon length, cecum weight and spleen weight). In addition, inflammation of the colon and cecum as well as hyperplasia was reduced. Zerumbone treatment significantly inhibited colonic polyp numbers and prevented macroadenoma progression. Taken together, these findings suggest that oral treatment with zerumbone inhibited ETBF-promoted colon carcinogenesis in mice indicating that zerumbone could be employed as a promising protective agent against ETBF-mediated colorectal cancer.

## 1. Introduction

Colorectal cancer (CRC) refers to a tumor of the large bowel that arises from the colorectal mucosa [1]. Adenocarcinoma is the most common form of colorectal cancer (>95%). Colorectal cancer usually develops from adenomatous polyps (adenoma) that undergo dysplastic changes to become cancerous (adenocarcinoma). The risk of CRC increases with the duration of colitis and extent of colonic inflammation. For example, patients with inflammatory bowel diseases (IBD) have an estimated 2–3-fold higher incidence of CRC than the general population [2]. It has been hypothesized that a key player in the development of CRC might be the bacterial commensal enterotoxigenic *Bacteroides fragilis* (ETBF). ETBF is a molecular subset of *Bacteroides fragilis* distinguished based on the secretion of a sole virulence factor, the *Bacteroides fragilis* toxin (BFT) [3,4]. The epithelial response to BFT shows E-cadherin cleavage resulting in NF-κB signaling in colonic epithelial cells [5,6,7,8,9].

Increased prevalence of ETBF has been reported in patients with IBD and those with CRC [10,11]. ETBF infection induces chronic colitis, which is characterized by rapid and robust colonic activation of the signal transducer and activator of transcription 3 (STAT3) protein, which subsequently results in a T-helper type 17 (Th17) immune response and leads to the evolution of colorectal cancer [12,13,14,15]. According to Sears’s group, ETBF infection in multiple intestinal neoplasia (Min) mice induces many adenomas in the distal colon [15]. Additionally, we have demonstrated that ETBF infection also promotes azoxymethane (AOM)/dextran sulfate sodium (DSS)-mediated colitis-associated cancer in wild-type mice [16]. ETBF infection in AOM/DSS-treated mice demonstrates the rapid formation of high-grade adenomas in the distal colon [16].

Clearance of ETBF by cefoxitin, a semi-synthetic and broad-spectrum cepha antibiotic, inhibited ETBF-promoted tumorigenesis in Min mice [17]. However, there exist no studies investigating the suppressive effects of natural product on ETBF-mediated tumorigenesis in mice. Previous research has demonstrated health-promoting properties of various dietary natural compounds, including carotenoids, flavonoids, and polyphenols contained in vegetables and fruits [18,19]. Chemoprevention is defined as the administration of dietary natural compounds and/or compounds chemically synthesized that prevents and/or delays the development of cancers. In the case of colon cancer, retarding the progression of adenoma to adenocarcinoma. Zerumbone is a main component of the subtropical ginger plant *Zingiber zerumbet*. Recent studies revealed several biological properties of zerumbone that may be responsible for inhibition of ETBF-induced colon carcinogenesis. Zerumbone has been reported to have anti-bacterial, anti-inflammatory and anti-cancer activities [19,20,21,22,23]. Zerumbone decreases ETBF infection-induced colon inflammation in mice via suppression of NF-κB signaling irrespective of ETBF colonization [9]. Inhibition of NF-κB signaling observed in colon of ETBF-infected mice administered zerumbone is directly BFT-dependent, since in vitro, zerumbone treatment diminishes NF-κB activation in BFT-exposed HT29/C1 human colon carcinoma cells [9]. As our initial findings demonstrated the suppression of colonic inflammation to ETBF infection by zerumbone, we hypothesized that zerumbone decreases ETBF-mediated tumorigenesis in mice. In the present work, we investigated whether oral treatment of zerumbone inhibits tumorigenesis in AOM/DSS mice colonized with ETBF.

## 2. Results

### 2.1. Experimental Scheme and Effects of Zerumbone on Body Weight and Disease Activity Index in ETBF/AOM/DSS Mice

**Figure 1:**
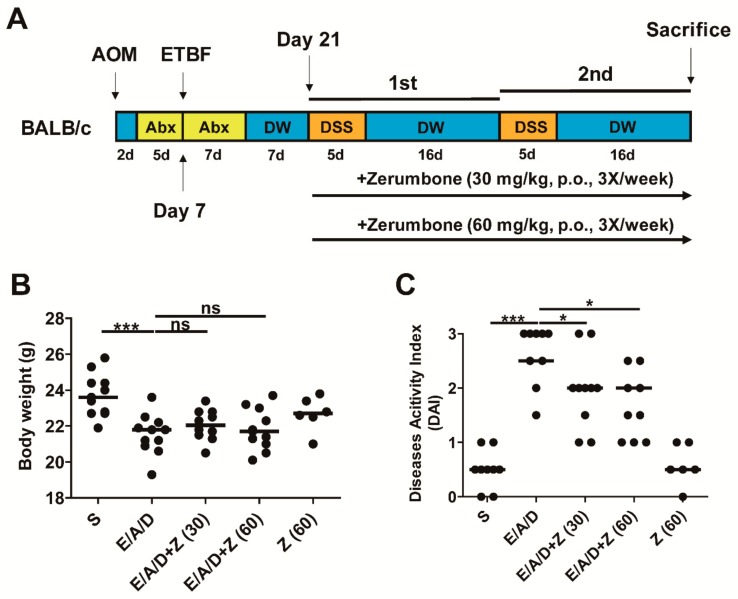
Experimental scheme and effects of zerumbone on body weight and the disease activity index in enterotoxigenic *Bacteroides fragilis* (ETBF)/AOM/DSS mice. BALB/c mice were given a single intraperitoneal injection of AOM (10 mg/kg) and provided drinking water ad libitum containing clindamycin/gentamicin for 5 days (yellow box). ETBF was orally inoculated and the antibiotic cocktail was continued for an additional 7 days (yellow box). Seven days later, BALB/c mice were subjected to two cycles of 1% DSS (5 days per cycle, orange boxes) and distilled water (DW; 16 days per cycle, blue boxes). During the two DSS cycles, BALB/c mice were given zerumbone (30, 60 mg/kg, p.o., three times a week). The total period of the experiment was 9 weeks. (**A**) Experimental design of the ETBF/AOM/DSS-induced tumorigenesis model. (**B**) Body weight. (**C**) Disease activity index (DAI). Body weight and DAI were measured at the last day of ETBF/AOM/DSS-treated experiments. S, sham control; E, ETBF; A, AOM; D, DSS; Z (30), Zerumbone (30 mg/kg) and Z (60), Zerumbone (60 mg/kg). Each dot represents one mouse (*n* = 6–12 mice per group). Scatter plot. Horizontal bar, median. * p < 0.05, *** p < 0.001. ns, no statistical significance.

In the ETBF/AOM/DSS model, mice exhibited an aggravated pathology manifested by colitis in association with body weight loss and clinical symptoms such as ruffled fur, lethargy and bloody diarrhea [16]. To assess the chemopreventive effect of zerumbone on the ETBF-mediated CRC, BALB/c mice were injected with AOM (10 mg/kg) once and provided with distilled water containing clindamycin and gentamicin 2 days later for a total of 12 days (Figure 1A). Subsequently, distilled water was provided for the entire duration of the experiment. Wild-type ETBF (1 × 10^9^ CFU) were orally inoculated once at day 7. At day 21, the first DSS cycle (5 days of 1% DSS + 16 days of distilled water) was initiated with a total of two DSS cycles. AOM/DSS mice colonized with WT-ETBF were administered zerumbone (30 or 60 mg/kg, p.o.) during two DSS cycles, three times a week (Figure 1A). Mice were sacrificed at the end of the second DSS cycle.

Decreased body weight has been positively associated with increased colonic inflammation in colitis-associated cancer (CAC) [24]. Therefore, to determine if zerumbone decreases ETBF/DSS-induced colonic inflammation, the body weight of BALB/c mice was measured during the two DSS cycles. During the ETBF/AOM/DSS-induced CAC experiment, no significant difference in the body weight between the ETBF/AOM/DSS group and the zerumbone-treated ETBF/AOM/DSS group was found (Figure 1B). Symptomatic parameters of colitis, such as activity, rectal bleeding, and severity of stool consistency, were scored from 0 to 3 to obtain the disease activity index (DAI; see Table A1). The DAI score also showed no alterations between ETBF/AOM/DSS group and zerumbone-treated ETBF/AOM/DSS groups during the first cycle of DSS (data not shown). However, after the second cycle of DSS, it was observed that ETBF/AOM/DSS groups treated with zerumbone (30 and 60 mg/kg) showed decreased DAI score as compared with the ETBF/AOM/DSS group (Figure 1C).

### 2.2. Zerumbone Reduced ETBF-Mediated Tumorigenesis in AOM/DSS Mice

**Figure 2:**
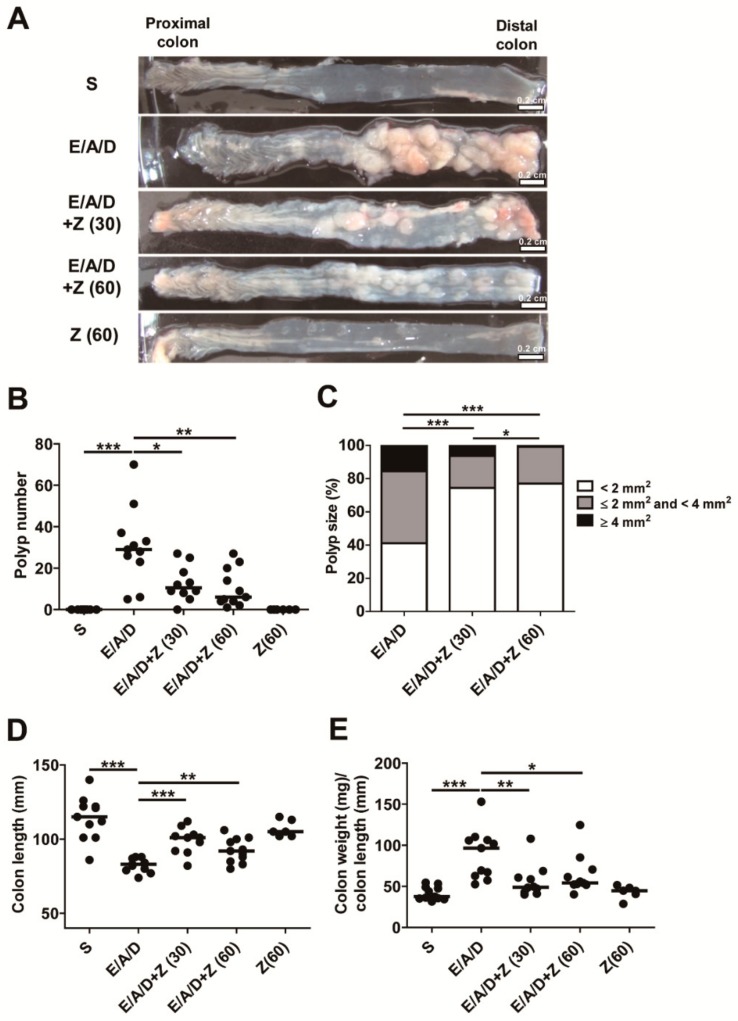
Zerumbone reduced ETBF-mediated tumorigenesis in AOM/DSS mice. AOM-treated BALB/c mice were infected with ETBF and subjected to two cycles of DSS (1%) for 9 weeks. During two DSS cycles, BALB/c mice were given zerumbone (30 and 60 mg/kg, p.o., three times a week). (**A**) Representative gross macroscopic image of the colon. (**B**) Polyp number. (**C**) Polyp size distribution. (**D**) Colon length (mm) and (**E**) colon weight (mg)/colon length (mm) ratio. Polyp number, polyp size distribution, colon length and colon weight/colon length ratio were measured on the last day of ETBF/AOM/DSS-treated experiments. S, sham control; E, ETBF; A, AOM; D, DSS; Z (30), Zerumbone (30 mg/kg) and Z (60), Zerumbone (60 mg/kg). Each dot represents one mouse. Scatter plot. Horizontal bar, median. * *p* < 0.05, ** *p* < 0.01, *** *p* < 0.001. ns, no statistical significance.

We next examined the impact of zerumbone on ETBF-mediated tumorigenesis in AOM/DSS mice. Results indicated that ETBF-colonized AOM/DSS mice administered with zerumbone (30 and 60 mg/kg) exhibited significant decreased polyp formation compared to ETBF-colonized AOM/DSS mice (Figure 2A,B). In addition, a change in the distribution of polyp sizes was seen, implying that tumors that form after zerumbone treatment may progress more slowly (Figure 2C). Specifically, the proportion of polyps in the 2–4 mm^2^ size range was decreased in ETBF-colonized AOM/DSS group administered with zerumbone (30 and 60 mg/kg; Figure 2C). As previously reported, ETBF infection promotes AOM/DSS-mediated tumorigenesis as well as enhanced colon inflammation, decreased colon length and increased colon weight/colon length ratio [16]. Consistent with the observation, we also found a decrease in colon length and increase in colon weight/colon length ratio in ETBF/AOM/DSS mice (Figure 2D,E). Furthermore, zerumbone treatment in ETBF/AOM/DSS mice increased colon length and decreased colon weight/colon length ratio compared ETBF/AOM/DSS mice (Figure 2E). Treatment of zerumbone alone in control mice exerted no effects on either colon length or colon weight/colon length ratio.

### 2.3. Zerumbone Reduced the Number of High-Grade Macroadenomas in ETBF-Colonized AOM/DSS Mice

**Figure 3:**
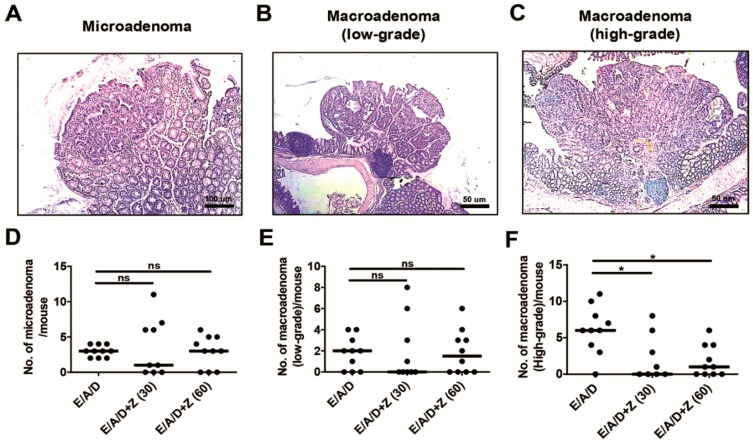
Zerumbone decreases ETBF colonization-promoted high-grade macroadenomas. FFPE colonic tissues of distal colon obtained from AOM-treated BALB/c mice infected with ETBF and subjected to two cycles of DSS (1%) for 9 weeks were excised and stained with H&E. A representative image of microadenoma, low-grade macroadenoma and high-grade macroadenoma is shown. Microadenoma, low-grade macroadenoma and high-grade macroadenoma were counted for two H&E-stained distal colon of sections per mouse. (**A**) Histology of microadenoma, ×100 magnification. (**B**) Histology of low-grade macroadenoma, 50× magnification. (**C**) Histology of high-grade macroadenoma, 50× magnification. (**D**) Number of microadenoma per mouse. (**E**) Number of low-grade macroadenoma per mouse. (**F**) Number of high-grade macroadenoma per mouse. E, ETBF; A, AOM; D, DSS; Z (30), Zerumbone (30 mg/kg) and Z (60), Zerumbone (60 mg/kg). Each dot represents one mouse. Scatter plot. Horizontal bar, median. * *p* < 0.05. ns, no statistical significance.

Microadenomas are adenomas that are not grossly visible, are characterized by dysplasia with pronounced nuclear atypia and have a loss of nuclear polarity [25]. Microadenoma formation precedes the formation of macroadenoma [25]. As zerumbone treatment led to a decrease in the number of polyps formed in the ETBF-colonized AOM/DSS group, we determined whether zerumbone treatment changes the initiation of microadenomas or their progression to low-grade macroadenomas or high-grade macroadenomas by counting the number of microadenomas, low-grade- and high-grade-macroadenomas, using a histopathologic analysis. We found that the number of microadenomas and low-grade macroadenomas per mouse were statistically insignificant between the ETBF/AOM/DSS and zerumbone treated ETBF/AOM/DSS treated groups (Figure 3A,B,D,E). However, the number of macroadenomas was decreased in zerumbone-treated ETBF/AOM/DSS treated mice compared to ETBF/AOM/DSS treated mice (Figure 3C,F). These results suggest that zerumbone inhibits primarily the initiation of adenomas but not the progression of polyp formation.

### 2.4. Zerumbone Decreased Large Intestinal Inflammation and Hyperplasia in ETBF-Colonized AOM/DSS Mice

**Figure 4:**
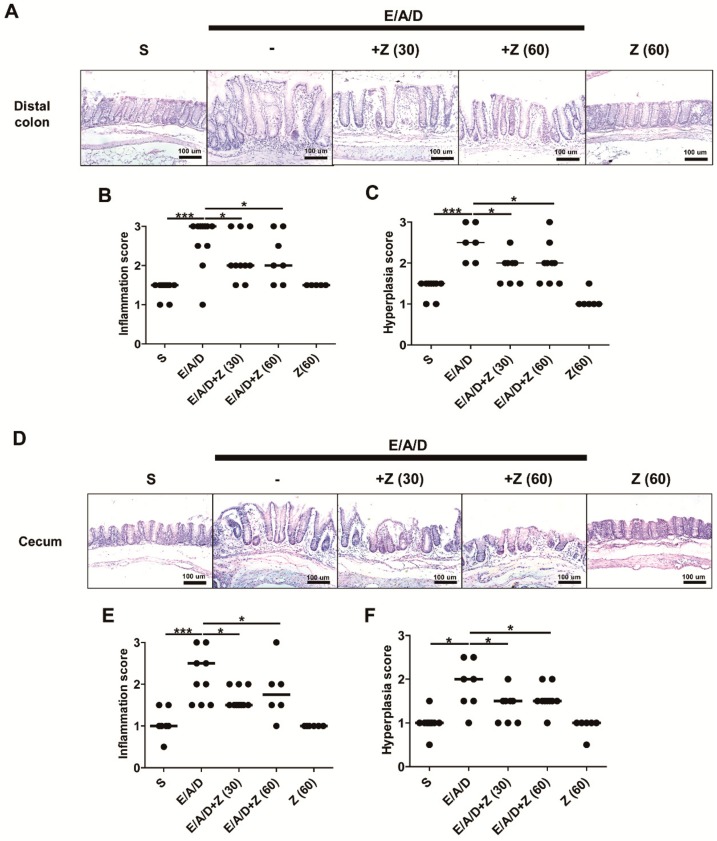
Zerumbone decreased large intestinal inflammation and hyperplasia in ETBF-colonized AOM/DSS mice. AOM-treated BALB/c mice were infected with ETBF and subjected to two cycles of DSS (1%) for 9 weeks. During two DSS cycles, BALB/c mice were administered with zerumbone (30 and 60 mg/kg, p.o., three times a week). (**A**) Histology (H&E) of distal colon tissues, 100× magnification. *B*. Inflammation score (distal colon). (**C**) Hyperplasia score (distal colon). (**D**) Histology (H&E) of cecal tissues, 100× magnification. (**E**) Inflammation score (cecum). (**F**) Hyperplasia score (cecum). Each dot represents one mouse. Scatter plot. Horizontal bar, median. S, sham control; E, ETBF; A, AOM; D, DSS and Z (60), Zerumbone (60 mg/kg). * *p* < 0.05, *** *p* < 0.001. ns, no statistical significance.

Enhanced tumorigenesis is positively associated with increased inflammation in tissues in a mouse model with inflammatory disorders [26]. In addition, reduced polyp formation and reduction on the level of indirect markers (i.e., colon length and colon weight/colon length ratio) prompted further analysis on the histology of distal colon obtained from BALB/c mice in all groups, followed by the evaluation of colonic inflammation and hyperplasia (Table A1). Results indicated decreased inflammation and hyperplasia in distal colon of ETBF-colonized AOM/DSS group administered with zerumbone (30 and 60 mg/kg) compared to ETBF-colonized AOM/DSS group (Figure 4A–C). Similarly, decreased inflammation and hyperplasia were observed in the cecum of ETBF-colonized AOM/DSS group administered with zerumbone (30 and 60 mg/kg) compared to ETBF-colonized AOM/DSS group (Figure 4D–F). These results strongly suggest that the decreased in polyps observed in the zerumbone treated ETBF/AOM/DSS mice was due to decreased colonic inflammation and hyperplasia.

### 2.5. Zerumbone Prevented Cecum Weight Loss and Spleen Enlargement in ETBF-Colonized AOM/DSS Mice

**Figure 5:**
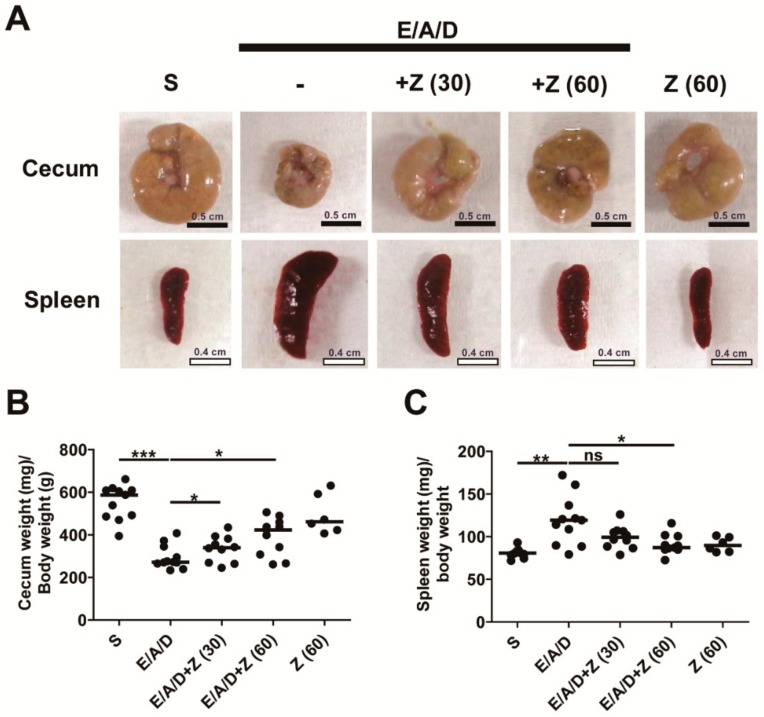
Zerumbone prevented cecum weight loss and spleen enlargement in ETBF-colonized AOM/DSS mice. AOM-treated BALB/c mice were infected with ETBF and subjected to two cycles of DSS (1%) for 9 weeks. During two DSS cycles, BALB/c mice were administered with zerumbone (30 and 60 mg/kg, p.o., three times a week). Mice were euthanized after two cycles of 1% DSS treatment. (**A**) Representative images of cecum and spleen. (**B**) Cecum weight. (**C**) Spleen weight. S, sham control; E, ETBF; A, AOM; D, DSS; Z (30), Zerumbone (30 mg/kg) and Z (60), Zerumbone (60 mg/kg). Each dot represents one mouse. Scatter plot. Horizontal bar, median. * *p* < 0.05, *** *p* < 0.001. ns, no statistical significance.

The decrease in the cecum weight with increased inflammation has been noted in ETBF murine models likely demonstrating a response to cecal injury [12]. Cecum weight is inversely associated with cecum inflammation in the ETBF murine model [12]. Consistent with these previous results, we found that the cecal weight of ETBF-colonized AOM/DSS group was decreased compared with the control AOM/DSS group (Figure 5A,B). ETBF-colonized AOM/DSS group administered with zerumbone (30 and 60 mg/kg) showed increased cecum weight compared with the ETBF-colonized AOM/DSS group (Figure 5A,B). Spleen enlargement is also positively associated with colon inflammation in mice infected with ETBF [12]. The spleen weight of the ETBF-colonized AOM/DSS group was increased compared with the sham control (Figure 5A,C). Whereas, zerumbone (60 mg/kg)-treated ETBF-colonized AOM/DSS group showed a marked decrease in spleen weight as compared with the ETBF-colonized AOM/DSS group (Figure 5C). Overall, these data are consistent with the results of colitis and tumorigenesis, suggesting that oral treatment with zerumbone may be beneficial as a preventative measure against ETBF-prompted colon inflammation and colorectal cancer.

### 2.6. Zerumbone Does Not Affect ETBF Colonization in AOM/DSS Mice

**Figure 6:**
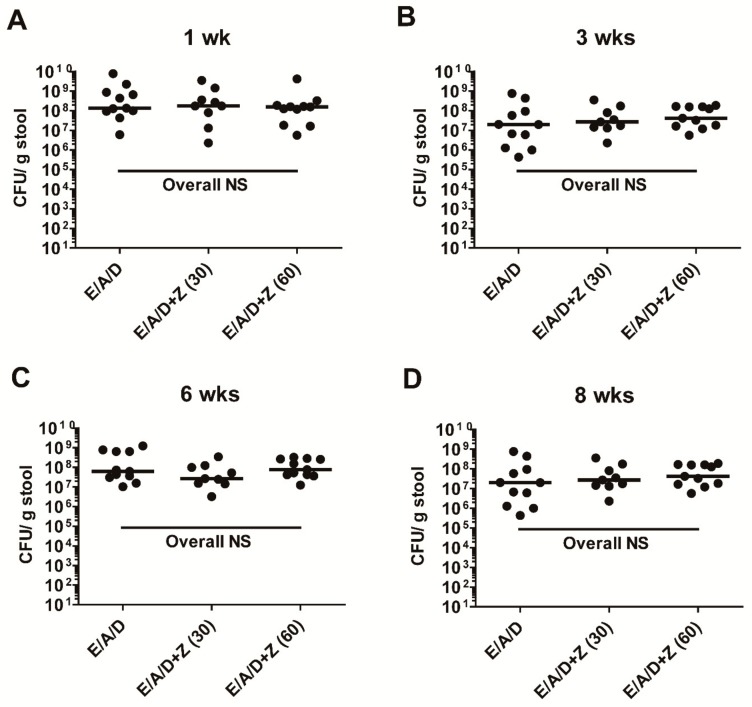
Zerumbone does not affect ETBF colonization in mice. ETBF colonization was assessed by stool plating. Colonization was generally within the range of 10^7^–10^9^ CFU/g stool at 1 (**A**), 3 (**B**), 6 (**C**) and 8 (**D**) weeks after ETBF infection. S, sham control; E, ETBF; A, AOM; D, DSS; Z (30), Zerumbone (30 mg/kg); Z (60), Zerumbone (60 mg/kg); CFU, colony-forming units and nd, not detected. Each dot represents one mouse. Scatter plot. Horizontal bar, median. ns, no statistical significance.

Earlier it has been reported that zerumbone exhibits antimicrobial effects against bacteria [20,23]. In addition, cefoxitin-induced ETBF clearance resulted in reduced tumorigenesis in Min^ApcΔ716^ mice [17], thus prompting us to investigate the number of ETBF in feces from ETBF-colonized AOM/DSS groups treated with zerumbone (30 and 60 mg/kg). We found that oral administration of zerumbone did not have any adverse effects on ETBF colonization in AOM/DSS mice during the length of the experiment (Figure 6). This result suggests that the protective effect of zerumbone is not due to the inhibition of ETBF colonization but rather on the host tissues.

## 3. Discussion

Previously, we demonstrated that ETBF infection promoted AOM/DSS-induced tumorigenesis in BALB/c mice with increased serum cytokines related to Th17 immune response [16]. However, there exist no clinical or animal studies investigating the potential natural products to prevent ETBF-promoted colonic tumorigenesis. In the present study, we demonstrated that BALB/c mice orally administered with zerumbone (30 and 60 mg/kg) exhibited decreased tumorigenesis promoted by ETBF colonization in AOM/DSS mice. Our key finding was that zerumbone (30 and 60 mg/kg)-treated ETBF/AOM/DSS group markedly decreased progression to visible macroadenomas by almost 3-fold compared to the ETBF/AOM/DSS group. In contrast, formation of low-grade macroadenomas was similar in both groups regardless of treatment with zerumbone. This data suggests that zerumbone treatment suppresses tumor progression promoted by ETBF infection. Since zerumbone did not decrease ETBF colonization in mice, the effect of zerumbone is likely due to chemopreventive effects on the host. In the current study, zerumbone treatment induced no alterations in ETBF number in the stool of ETBF-colonized AOM/DSS model, but suppressed ETBF-mediated tumorigenesis. This result is consistent with our previous findings and showing that ETBF infected mice given zerumbone show no change in ETBF colonization [9]. The addition of AOM/DSS in the current study also did not affect ETBF colonization density.

The key ETBF virulence factor is *Bacteroides fragilis* toxin (BFT), a zinc-dependent metalloprotease that targets epithelial adherence junctions [7]. In vitro studies using the human colonic epithelial cell line, HT29/C1, showed that BFT induces ectodomain cleavage of E-cadherin resulting in the loss of the epithelial barrier function and activation of the β-catenin pathway. In vivo, catalytically active BFT also cleaves E-cadherin accompanied by colonic inflammation in mice [12]. Our previous study showed that zerumbone-treated colonic epithelial cells down-regulated NF-κB signaling and IL-8 expression in response to BFT exposure [9]. However, zerumbone treatment does not inhibit BFT-induced E-cadherin cleavage in colonic epithelial cells in HT29/C1 cells [9]. We speculate that zerumbone might suppress ETBF-promoted tumorigenesis in AOM/DSS mice, in part by, via down-regulation of NF-κB signaling induced by E-cadherin cleavage [27,28].

A number of studies have shown direct anti-cancer effects of zerumbone numerous cancer cell lines derived from the breast, brain, liver, lung and colon (reviewed in [29]). Zerumbone modulated various proteins and signaling pathways including the IL-6/JAK2/STAT3 and NF-κB pathways and the associated pro-carcinogenic genes such as *IL-6*, *COX2* and *cyclin D1*, thus inhibiting cellular proliferation and promoting apoptosis. Regarding zerumbone effects on colorectal cancer cell lines, zerumbone decreased expressed of cFLIP, which in turn stimulated TRAIL death receptor DR4 and DR5, augmenting TRAIL-induced apoptosis in HCT116 colon cancer cell line [22]. The zerumbone treatment also leads to cell cycle arrest and increased apoptosis in three colorectal cell lines (HCT116, SW620 and HT29) [30]. Mice injected with AOM and given DSS, oral zerumbone treatment for 17 weeks resulted in decreased colon inflammation and colon polyp formation through inhibition of NF-κB and heme oxygenase-1 [31].

A recent study by Dermani et al. showed that zerumbone treatment of HCT116 and SW48 colorectal cell lines inhibited cell viability, migration, invasion and sphere-forming potential via miR-200c [32]. Silencing of miR-200c reduced the anti-cancer effects of zerumbone. Of interest, zerumbone suppressed β-catenin, which plays a prominent role in colorectal cancer progression in the ETBF model. This result suggests that zerumbone may not only target the STAT3 pathway but also the β-catenin pathway as well. ETBF-induced tumorigenesis is dependent on IL-17A cytokines secreted by both Th17 and γδ T cells [13]. In the course of ETBF infection, early pro-tumorigenesis is dependent on Th17-derived IL-17A but late pro-tumorigenesis is chiefly mediated by γδ T cell-derived IL-17A in ETBF-infected Min mice [13]. A recent report by Jantan et al., performed a comprehensive analysis of both the innate and adaptive immunes responses in BALB/c mice orally administered zerumbone for 14 days [33]. They found a decrease in phagocytosis by peritoneal macrophages and subsequent release of NO and MPO in a concentration-dependent manner. Zerumbone also suppressed T lymphocyte proliferation and Th1/Th2 cytokine release. Although, effects of zerumbone on Th17 cytokine release was not examined, the pan-specific inhibition of both Th1/Th2 cytokines suggest that Th17 cytokines were also inhibited.

In summary, we report that oral administration of zerumbone diminishes ETBF-mediated tumorigenesis in BALB/c mice via inhibition of colonic inflammation. Expanding upon our previous studies, we found that zerumbone does not affect ETBF colonization but rather ameliorates colonic inflammation, which in turn decreases colorectal cancer. The effects of zerumbone in the ETBF infected AOM/DSS mice is potentially multifactorial—affecting the Th17 cells, β-catenin pathway, STAT3 pathway and the NF-κB pathway. When translated into the clinical setting, individuals harboring ETBF could potentially benefit from consumption of zerumbone-containing foods.

## 4. Materials and Methods

### 4.1. Bacteria Strains and Bacteria Enumeration in Stool

The wild-type ETBF strain used in the current study was *B. fragilis* 86-5443-2-2 (*bft-2*). The wild-type *Bacteroides fragilis* strain is resistant to gentamicin and clindamycin. Colonization of bacteria in mouse stool was monitored by serial dilution and plating of stool on brain heart infusion agar (BHIA) plates containing 50 μg/mL of gentamicin (Corning, New York, NY, USA) and 6 μg/mL of clindamycin (Hospira, Chicago, IL, USA). Plates were incubated overnight at 37 °C under anaerobic conditions (Mitsubishi Gas Chemical Company, Tokyo, Japan). The addition of clindamycin and gentamicin to the BHIA plates prevents growth of other fecal bacteria allowing enumeration of the wild-type *Bacteroides fragilis.* Characteristic *B. fragilis* colonies were enumerated after anaerobic culture and shown as colony-forming units (CFU)/gram stool [12]. The bacterial strain was a generous gift from Cynthia Sears (Johns Hopkins University, Baltimore, MD, USA).

### 4.2. ETBF/AOM/DSS Mouse Model

All animal housing and experimental procedures were reviewed and approved by the Institutional Animal Care and Use Committee of Yonsei University MIRAE Campus (YWCI-201901-002-01, approval date: 23 January 2019. All experiments were performed to conform to relevant guidelines and regulations under the Institutional Animal Care and Use Committee of Yonsei University MIRAE Campus. Eight-week-old female BALB/c mice (Rion-bio, Yongin, Korea) received a single intraperitoneal injection of 10 mg/kg of AOM (Sigma-Aldrich, St. Louis, MO, USA). Two days later, mice were given water containing clindamycin (100 mg/L) and gentamicin (300 mg/L) for 5 days to promote colonization of *B. fragilis*. Mice were inoculated with bacteria and antibiotic-containing water was continued for an additional 7 days. After 7 days of distilled water (DW) administration, the first DSS cycle was initiated (5 days DSS, 16 days of DW) for a total of three cycles. DSS (36–50 kDa) was purchased from MP Biomedicals (Irvine, CA, USA). Bacteria were grown in brain heart infusion broth (Becton Dickinson, Franklin Lakes, NJ, USA) and adjusted to 1 × 10^9^ CFU/200 μL for mouse oral inoculations.

### 4.3. Tumor Enumeration and Histopathology

Macroscopic tumors were counted after sacrificing the mice. An unstained large intestine was examined by one investigator using a stereomicroscope to count the polyp number and size. The length and width of the polyp were measured by a microcaliper. The polyp size was calculated as length × width. Polyps were grouped as < 2 mm^2^, 2–4 mm^2^ and > 4 mm^2^. Results are presented as the median polyp size. For histologic studies including microadenoma, low-grade macroadenoma and high-grade macroadenoma quantification, formalin-fixed tissue was paraffin-embedded, sectioned (5 μm), and stained with hematoxylin and eosin (H&E). Microadenoma, low-grade macroadenoma and high-grade macroadenoma were examined by two investigators. Microadenoma, low-grade macroadenoma and high-grade macroadenoma were examined for two H&E-stained sections of distal colon per mouse, and quantified in representative 20 randomly selected 200× field per specimen. Representative images were taken using an optical microscope and rendered using Adobe Photoshop (Adobe, San Jose, CA, USA).

### 4.4. Zerumbone Treatment in ETBF/AOM/DSS Mice

Zerumbone was purchased from Kingherbs (Hunan, China). BALB/c mice were given AOM once (10 mg/kg), ETBF (1 × 10^9^ CFU/200 μL) and two cycles of DSS (1%). Administration of zerumbone was initiated simultaneously with the DSS treatment. During two cycles of DSS, BALB/c mice were treated with zerumbone (30 and 60 mg/kg, p.o.) three times a week. Mice from each of the groups were monitored daily for water consumption.

### 4.5. Evaluation of Inflammation and Hyperplasia

To facilitate longitudinal examination of the full-length colon, colons were “Swiss-rolled” prior to embedding and sectioning. Sections were stained with H&E and observed for histological assessment in terms of severity of inflammation, extent of injury, regeneration and crypt damage and proliferation of epithelial cell. The final inflammation or hyperplasia score was calculated by the sum of the scores for all parameters. Colon inflammation was evaluated as follows: 0, normal; 1, mild increase in immune cells and no colonic epithelial alterations; 2, a moderate increase in immune cells and mild colonic epithelial proliferation and 3, severe increase in immune cells and aberrant colonic epithelial proliferation with extensive loss of crypt architecture. Hyperplasia was graded as follows: 0, normal; 1, mild increase in crypt length; 2, moderate crypt length with hyperchromatic colonic epithelial cells and 3, severe increase in crypt length with high mitotic index. Representative images were taken using an optical microscope and rendered using Adobe Photoshop.

### 4.6. Statistics

Comparison of the median was done using the unpaired, two-tailed Mann–Whitney *U* test unless otherwise indicated. Statistical analyses were performed using GraphPad Prism (GraphPad Software Inc., La Jolla, California, USA). A *p* value of < 0.05 was considered to indicate a statistically significant difference.

## Appendix A

**Table A1:** Scoring of the disease activity index (DAI).

DAI Score	Activity	Stool Consistency	Occult/Gross Bleeding
0	Normal	Normal	Normal
1	Moderately decreased activity	Slightly loose stool	Slightly hemoccult positive in stool
2	Severely decreased activity	Severely Loose stool	Hemoccult positive in stool and rectum
3	None	Diarrhea	Gross bleeding
